# Antibacterial Activity of Bacteriophage Lytic Enzyme Ply900

**DOI:** 10.3390/vetsci13010065

**Published:** 2026-01-09

**Authors:** Yuan Li, Luxiang Xu, Yuhan Zhang, Chunliu Dong, Han Zhou

**Affiliations:** College of Veterinary Medicine (Preventive Veterinary Medicine), Northeast Agricultural University, No. 600, Changjiang Road, Xiangfang District, Harbin 150030, China; 13181390016@163.com (Y.L.); a1132701069@163.com (L.X.); zhangyuhan279@163.com (Y.Z.)

**Keywords:** *Streptococcus suis*, bacteriophage lytic enzyme, antibiotic replacement, multi-drug resistance

## Abstract

*S. suis* is a prominent zoonotic pathogen responsible for diseases such as arthritis in piglets, swine septicemia, and meningitis. The emergence of multi-drug resistance (MDR) underscores the urgent need for the development of novel antibacterial strategies. In this context, a systematic evaluation of the antibacterial potential of the bacteriophage lytic enzyme Ply900 was conducted in this study, along with an analysis of its domain functions and an in vivo study of its therapeutic dynamics. Ply900 exhibits potent in vitro lytic activity against multiple bacteria, including *Streptococcus suis*, *Streptococcus agalactiae*, and *Staphylococcus aureus*. Notably, it possesses broad biochemical stability, with tolerance to diverse environmental conditions. In a mouse model of *S. suis* serotype 2 SC19 infection, both the direct Ply900 treatment group and the triple therapy group achieved effective eradication of *S. suis*, with markedly improved survival rates. The remaining bacteria remained susceptible to Ply900, with no evidence of induced resistance development. Mechanistic analysis revealed that the SH3B domain of Ply900 enhances targeted cleavage efficiency by binding synergistically to peptidoglycan with the CHAP domain, with CYS-34, HIS-59, and ASP-28 serving as key amino acid sites for Ply900’s cleavage activity. Collectively, these findings lay the foundation for the potential dual applications of the lysin Ply900, both in the clinical treatment of *S. suis* infections and in the prevention and control of these pathogenic bacteria in livestock farming.

## Simple Summary

This study identified a novel bacteriophage lytic enzyme, Ply900, which exhibits strong lytic activity against various serotypes of *Streptococcus* in vitro. In the in vivo experiments, this enzyme exhibits protective effects in the mice challenged with lethal doses of *Streptococcus suis* (*S. suis*) serotype 2. The experimental results of this study suggest that lysozyme Ply900 can be classified as a potential antibacterial agent capable of effectively treating *S. suis* type 2 infections, demonstrating significant potential as an alternative to antibiotics.

## 1. Introduction

*S. suis* is a globally prevalent zoonotic pathogen responsible for endocarditis, septicemia, and pneumonia in both pigs and humans [1,2]. Capsular polysaccharide typing has identified 29 serotypes of this species, with serotypes 2 (SS2) and 9 (SS9) exhibiting the highest prevalence in pig populations [3,4]. *S. suis* serotype 2 is a highly pathogenic serotype and one of the primary pathogens responsible for zoonotic diseases and bacterial septicemia in swine populations worldwide. Owing to the misuse of antibiotics, the issue of MDR in the clinically isolated SS2 strains has become increasingly severe, posing a significant challenge to the treatment of SS2 infections globally [5]. Horizontal transfer of drug resistance genes is the key mechanism driving multi-drug resistance (MDR). Mobile genetic elements (e.g., plasmids, transposons) harbor multiple resistance genes, including ermB and tetM with an extremely high detection rate (over 80%), as well as sul2, pbp2x, and gyrA [2,6,7].

Only one virulent bacteriophage targeting *S. suis*, SMP (family Siphoviridae, genus Lambdavirus, ICTV 2022 taxonomy; strain no.: CGMCC 19876), has been reported to date. The narrow lytic spectrum of this bacteriophage limits its practical application in production, as it can only exert strong lytic activity against *S. suis* serotype 2 and exhibit weak cross-lytic activity against other serotypes or even none at all [8]. In this context, analyzing prophage sequences within the genomes of lysogenic bacteria to obtain highly efficient, broad-spectrum lytic genes serves as a crucial strategy for addressing the scarcity of virulent bacteriophages [9]. Phage lytic enzymes are novel targeted antibacterial agents that offer multiple advantages in several aspects, including potent bactericidal activity and suppression of drug resistance. The underlying mechanism involves the enzyme disrupting the double-layer structure of teichoic acids and peptidoglycan, thereby efficiently targeting and eliminating the target bacteria. Compared with antibiotics, phage lytic enzymes effectively prevent the expression of bacterial resistance genes while minimizing the risk of triggering systemic host immune responses, thereby ensuring therapeutic safety [6,10,11].

In this study, *S. suis* and *Streptococcus parasuis* strains were isolated and identified, which were derived from synovial fluid, blood, and visceral tissues of diseased pigs in pig farms located in the three northeastern provinces of China (Heilongjiang, Jilin, and Liaoning provinces), among which the most drug-resistant strain, *Streptococcus parasuis* (SS-HG-1010), was selected for further analysis, yielding the bacteriophage lytic enzyme-encoding gene Ply900. This study aims to characterize the biochemical properties of the phage lytic enzyme Ply900, elucidate its lytic mechanism by analyzing key domains (CHAP, SH3B) and critical amino acid mutations, evaluate its broad-spectrum lytic activity against *S. suis*, *Streptococcus agalactiae* and *Staphylococcus aureus*, assess its efficacy in eradicating *S. suis* biofilms and potential as an environmental disinfectant, and investigate its in vivo therapeutic effect against *S. suis* serotype 2 infection.

## 2. Materials and Methods

### 2.1. Strains and Culture Conditions

All *S. suis* strains used in the experiments were isolated from clinical specimens via inoculation on blood agar plates (37 °C, 5% CO_2_), identified using the species-specific *gdh* gene (glutamate dehydrogenase), and preserved in the laboratory. All *S. suis* strains were cultured aerobically in THB (Todd-Hewitt Broth, Sangon Biotech, Shanghai, China), broth at 37 °C inside a shaking incubator (220 rpm) overnight for 16 h. The bacterial load of *S. suis* was determined at 37 °C under static conditions using the THB agar supplemented with 5% horse serum [12]. The *Escherichia coli* (DH5α, BL21-DE3, Sangon Biotech) bacteria used for gene cloning and recombinant protein expression were cultured in LB (Luria–Bertani Broth, Sangon Biotech) medium at 37 °C inside a shaking incubator at 220 rpm.

### 2.2. Identification and Analysis of the Bacteriophage Lytic Enzyme Ply900

The prediction of the pre–phage for *Streptococcus parasuis* was performed using PHASTER (http://phaster.ca/instructions, accessed on 12 September 2024).

BLASTP and Phyre2 (http://www.sbg.bio.ic.ac.uk/phyre2, accessed on 21 December 2024) were employed to retrieve the phage lysozyme and predict the three-dimensional structure of Ply900. SMART (http://smart.embl-heidelberg.de/, accessed on 19 February 2025) was utilized to predict the major components of Ply900.

### 2.3. Expression and Purification of the Bacteriophage Lytic Enzyme Ply900

The gene encoding the bacteriophage lytic enzyme Ply900 (full genetic information pertaining in the Supplementary Materials.) was amplified from *S. suis* SS20 using design-specific primers (Ply900-R:ggaattccatatgctatttaaacgtaccgtaaggcac; Ply900-F:ccgctcgagatgacaacagtaaatgaagtagttaat). The Ply900 gene was then cloned and inserted into the pcold expression vector using homologous recombination, resulting in the pcold-Ply900 plasmid. The constructed plasmid was subsequently transformed into *E. coli* BL21-DE3 (Sangon Biotech). The culture was amplified at 37 °C until an OD600 (optical density at 600 nm) value of 0.4–0.5 (measured using an ALLSHENG-Nano-300 micro-spectrophotometer)was reached. After cooling, induction was performed with 0.5 mM IPTG (isopro-pyl-β-D_thiogalactopyranoside) at 16 °C for 18 h to express the phage lytic enzyme Ply900. Protein purification was then performed using a Ni-NTA (nickel-nitrilotriacetic acid) affinity chromatography column [13], followed by ultrafiltration tube dialysis to concentrate the protein. The protein concentration was then determined using a BCA (Bicinchoninic Acid) Protein Assay Kit (Thermo Fisher Scientific, Waltham, MA, USA).

### 2.4. Structural Analysis of the Ply900 Domain in Bacteriophage Lysin

Domain prediction of the phage lytic enzyme Ply900 was performed using InterPro, and its three-dimensional structure was modeled with SWISS-MODEL. Two fragments, Pcold-Ply900-CHAP and Pcold-Ply900-SH3B, were constructed, expressed, and purified. Two additional fragments, Pcold-Ply900-EGFP-CHAP and Pcold-Ply900-EGFP-SH3B, were constructed, expressed, and purified. The functionality of the CHAP domain was validated using turbidity reduction assays and laser confocal microscopy. Clusters were predicted using Clustal Omega (https://www.ebi.ac.uk/jdispatcher/msa/clustalo/summary?jobId=clustalo-I20250310-032331–0566-29178586-p1m&js=pass, accessed on 12 September 2024) to predict the pocket structure of Ply900. The spatial distribution was further validated using the structural visualization tool PyMOL (version 2.5.0), which identified residues CYS-34, HIS-56, HIS-61, HIS-89, ASP-28, ASP-83, and GLU-59. Targeted mutagenesis experiments then validate whether these residues constituted the key amino acid sites responsible for the cleavage effect. The lytic capabilities of seven purified Ply900 mutants were validated using plaque assays and turbidity reduction experiments.

### 2.5. Lysis Spectrum Determination In Vitro for the Bacteriophage Lytic Enzyme Ply900

The lysis spectrum of the lysis enzyme Ply900 was determined using the turbidity reduction method [14]. The strains used to determine the host range of Ply900, including *S. suis*, *Streptococcus agalactiae*, and *Staphylococcus aureus*, are listed in Table 1. Bacterial suspensions presented in Table 1 were prepared by mixing 100 μL of bacterial culture with 100 μL of Ply900 (20 μg/mL) in a 96-well plate, followed by incubation at 37 °C for 60 min. PBS (Phosphate-Buffered Saline, Sangon Biotech, Shanghai, China) served as the negative control, and the optical density at 600 nm was measured using a microplate reader (Spark, Tecan Austria GmbH, Grödig, Austria).

### 2.6. Determination of the Minimal Effective Concentration (MEC) of Ply900

The minimal effective concentration of the enzyme was determined [15]. Resuspension solutions of each bacterial strain from the lysis spectrum were prepared. Of the prepared bacterial suspension, 100 µL was added to the wells of a 96-well plate, and 100 μL of the lysis enzyme Ply900 was added to the bacterial suspension to achieve final concentrations of 40, 20, 15, 10, 5, and 1 μg/mL. The plates were incubated at 37 °C. Before measurement, the plates were shaken for 2 s at an amplitude of 2 mm. The OD600 values were recorded every 15 min, and changes in the OD600 over 60 min were documented to determine the MEC of Ply900. In all bacteriolytic activity assays for Ply900 using bacterial suspensions, cultures were adjusted to an OD600 = 0.6 prior to experimentation. Subsequent data normalization defined this turbidity level as 100% relative activity (baseline control).

### 2.7. Experimental Exploration of the Biochemical Characteristics of Bacteriophage Lytic Enzyme Ply900

The pH stability, thermal stability, salt ion stability, EDTA stability, and stability of the bacteriophage lytic enzyme Ply900 in piglet serum samples were evaluated using the turbidimetric assay. All assays were performed with a uniform enzyme concentration of 20 µg/mL (achieved by mixing Ply900 with resuspended bacterial suspension), and bacterial lysis was monitored by measuring the optical density at 600 nm (OD_600_) after co-incubation for 60 min. For pH stability, PBS buffers with gradient pH values (2–11) were prepared by adjusting with 1 mol/L HCl and 1 mol/L 9. Ply900 was pre-incubated with these buffers for 60 min, with pH confirmed before and after incubation using a calibrated precision meter. Bacterial suspensions were then added to the pre-treated enzyme for co-incubation. For thermal stability, Ply900 was pre-incubated at gradient temperatures (4, 16, 23, 26, 37, 45, 50, 55, and 60 °C) for 60 min, followed by the addition of bacterial suspensions for co-incubation under the same thermal conditions. For salt stability, the phage lytic enzyme Ply900 was incubated with the PBS buffer containing 1 M sodium chloride to achieve the final salt concentrations of 0, 50, 100, 150, 300, 400, 500, 750 mM, and 1 M. For EDTA stability, the phage lytic enzyme Ply900 was subjected to incubation with 0.1 M and 0.5 M EDTA (resuspended in PBS) at 37 °C for 60 min. In order to assess stability in piglet serum, the phage lytic enzyme Ply900 was incubated with healthy piglet serum for 0.5 h, 1 h, 2 h, 6 h, 12 h, 18 h, 24 h, 36 h, or 48 h.

### 2.8. Determination of the Effects of Bacteriophage Lytic Enzyme Ply900 on Biofilms

The pyrolysis spectrum of Ply900, *S. suis* serotype 9, exhibiting the strongest biofilm formation capacity, was selected to validate the inhibitory and removal effects of Ply900 on biofilms. The SS9 OD600 value of the shaken culture was 0.6. In a 96-well plate, 100 μL of the bacterial suspension and 100 μL of Ply900 were added to the wells of a 96-well plate, followed by incubation at 37 °C for 24 h, 48 h, or 72 h. In another process, 100 μL of bacterial suspension was added and incubated under the same conditions. Subsequently, 100 μL of Ply900 was added to the wells to achieve a concentration of 20 μg/mL, followed by incubation at 37 °C for 1 h. The inhibitory and removal effects of Ply900 on bacterial biofilm formation were assessed using 0.1% crystal violet staining and measurement of optical density at 590 nm with a microplate reader [14].

### 2.9. Mouse Model of S. suis Infection

Twenty-two-day-old female BALB/c mice weighing about 17 g were selected for establishing an *S. suis* infection model. *S. suis* serotype 2 was activated in the THB medium and cultured at 37 °C inside a shaking incubator until an OD600 of 1 was reached. The culture was then washed three times with sterile PBS, and each wash was followed by centrifugation at 5000× *g* for 5 min at 4 °C using a Thermo Fisher Scientific centrifuge. The bacterial suspension was adjusted to an OD600 of 1 using sterile PBS. A total of 96 mice were randomly divided into 4 groups (24 mice per group), with 12 mice in each group allocated to survival rate testing and the other 12 to bacterial load detection, namely, the Blank group, Control group, Direct treatment group, and Triple combination therapy group.

The mice in the direct treatment group were injected with a lethal dose of 8 × 10^8^ CFU of *S. suis* suspension. After a systemic infection was applied within 1 h, the phage lytic enzyme Ply900 200 μg (500 μg/mL) was administered through an unilateral intraperitoneal injection into the contralateral side. The triple therapy group received lethal doses of 8 × 10^8^ CFU *S. suis* suspension, followed by Ply900 treatment (70 μg per dose) at 6, 12, and 24 h post-infection. The control mice received a lethal dose of 8 × 10^8^ CFU *S. suis* suspension in one flank, with an equal volume of PBS buffer injected into the contralateral flank. Blank mice received an equal volume of PBS in both flanks.

The mice in the direct treatment group and triple therapy group were observed for 7 days post-infection. In the direct treatment group, three mice were randomly selected at 1 h, 12 h, 24 h, 36 h, and 48 h. In the triple therapy group, three mice were randomly selected at 12 h, 24 h, and 48 h post-treatment. Blood samples were collected, and the hearts, livers, spleens, lungs, and kidneys of the mice were examined to determine organ-specific bacterial loads. Serum samples were analyzed to measure the changes in inflammatory factors.

### 2.10. Data Statistics and Analysis

The experimental data were processed using SPSS 24.0 and GraphPad Prism 10.6. The data are presented as the means ± standard deviations (means ± SD). Intergroup differences were analyzed by one-way and two-way analysis of variance (ANOVA). A *p*-value less than 0.05 was considered statistically significant.

### 2.11. Ethical Approval and Animal Welfare

All animal experiments were approved by the Animal Ethics Committee of Northeast Agricultural University (Approval No.: NEAUEC20240345) and conducted in strict compliance with the Guidelines for the Welfare and Ethical Review of Laboratory Animals issued by the Ministry of Science and Technology of the People’s Republic of China (GB 14925-2010; Laboratory Animal Requirements of Environment and Housing Facilities; China Standards Press, Beijing, 2010).

## 3. Results

### 3.1. Function of the Phage Lytic Enzyme Ply900 Domain

**Figure 1:**
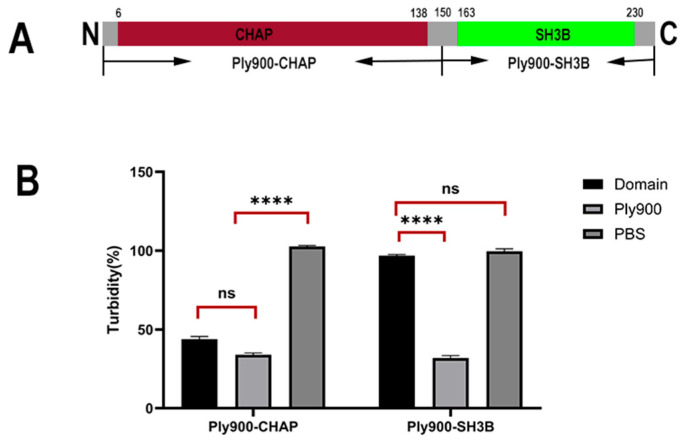
(**A**) Schematic Diagram of the Domains of Bacteriophage Lysin Ply900. (**B**) Lytic activity of truncated Ply900 expressing the CHAP and SH3B domains. Significant inter-group differences (ns *p* > 0.05, **** *p* < 0.0001). All experiments were repeated three times; error bars denote SD.

**Figure 2:**
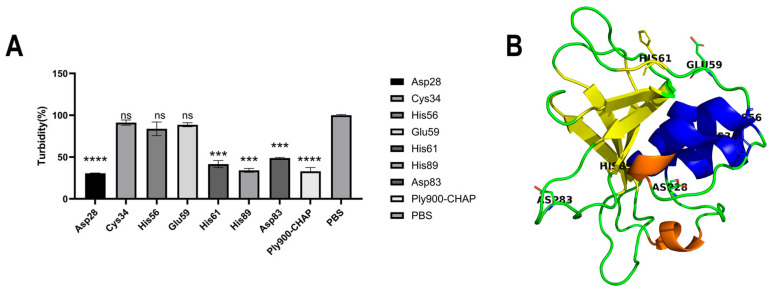
(**A**) Bactericidal activity of Ply900-CHAP and mutant proteins. (**B**) 3D structure of the Ply900-CHAP domain and schematic diagram of mutation sites. All experiments were replicated three times; error bars denote SD. (ns *p* > 0.05, *** *p* < 0.001, **** *p* < 0.0001).

**Figure 3:**
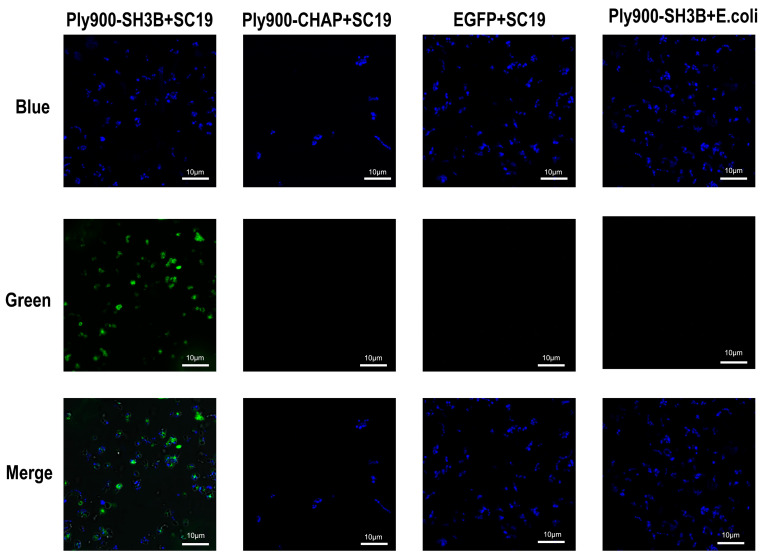
Laser confocal microscopy observation of different fusion proteins interacting with the bacterial cell wall. (Blue: represents viable bacteria stained with Hoechst 33342, imaged with blue excitation light at 405 nm. Green: represents EGFP fluorescence, imaged with green excitation light at 488 nm. Merge: combined blue and green channels showing the colocalisation of each fusion protein and EGFP with the bacterial strain.

The ability of the bacteriophage lytic enzyme Ply900 to lyse *S. suis* suggests that the domains constituting Ply900 possessed both lytic and cell wall-binding capabilities against *S. suis*. The bacteriophage lysin Ply900 comprises two distinct domains: a catalytic CHAP domain and a cell wall-binding SH3B domain (Figure 1A). Experimental results from point mutation validation at the key amino acid sites indicated that the lytic activity of the mutated enzymes Ply900-CYS-34, Ply900-HIS-56, and Ply900-GLU-59 decreased by more than 90%, whereas Ply900-HIS-61 and Ply900-ASP-83 mutations reduced the cleavage effect by about 40%, and Ply900-HIS-89 and Ply900-ASP-28 mutations retained nearly complete cleavage capacity (Figure 2A). Truncation experiments showed that the isolated CHAP domain retained lytic activity, with its efficacy reaching 88.73% compared to the full-length enzyme. In contrast, the SH3B domain alone exhibited no detectable lytic activity (Figure 1B). The SH3B binding domain, fused with an EGFP tag, demonstrated binding activity to the *S. suis* cell wall and exhibited specificity for *S. suis*, failing to bind to *E. coli* (Figure 3).

### 3.2. Bacteriophage Lytic Enzyme Ply900 Has a Broad Lytic Spectrum

**Figure 4:**
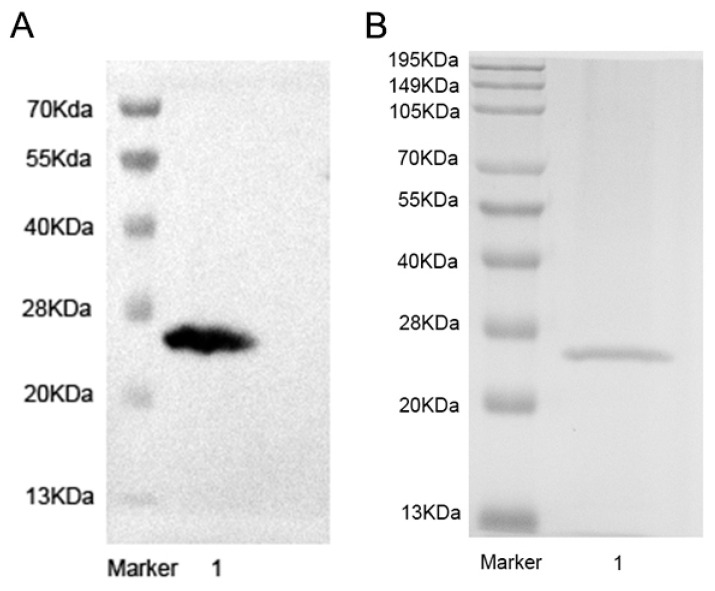
(**A**) Western blot analysis of recombinant His-tagged phage lysin Ply900; (**B**) SDS-PAGE analysis of purified recombinant Ply900 (200 μg). The complete blot image is shown in Supplementary Figure S1.

**Figure 5:**
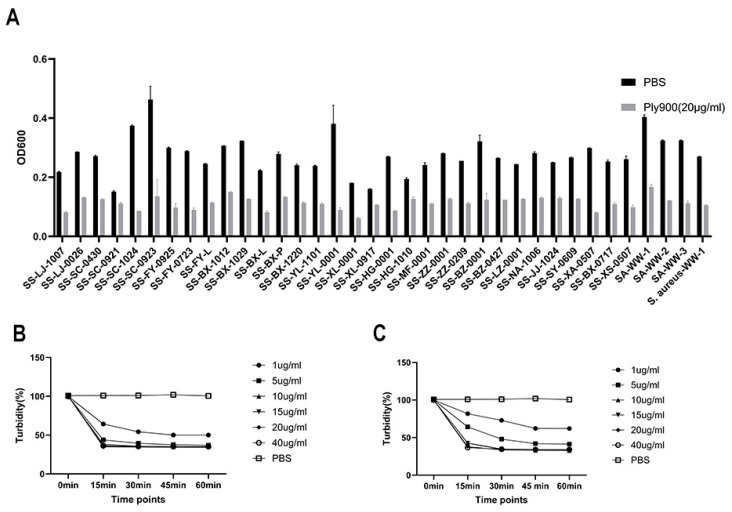
(**A**) Lysis spectrum of Ply900. (**B**) Lytic activity of Ply900 against *S. suis* SC19 at different concentrations. (**C**) Lytic activity of Ply900 against *Streptococcus agalactiae SA-WW-1* at different concentrations. All experiments were replicated three times; error bars denote SD.

The successfully expressed bacteriophage lytic enzyme Ply900 (Figure 4) exhibited high lytic activity against 31 *S. suis*, 3 strains of *Streptococcus agalactiae*, 1 strain of *Streptococcus parasuis* and 1 strain of *Staphylococcus aureus* (Figure 5A). Lytic efficiency demonstrated a marked dose-dependent relationship, with the initial lysis rate decreasing as the concentration of Ply900 decreased. At the lysis endpoint, viable bacterial counts reached 1.1 × 10^3^ CFU/mL for SC19 and 8.08 × 10^3^ CFU/mL for SA-WW-1. However, lysis reached its endpoint within 60 min in all cases with minimal replicate variability (Figure 5B,C). The Minimal Effective Concentrations of the enzymes in vitro are presented in the table below (Table 1).

### 3.3. Biochemical Characteristics of the Bacteriophage Lytic Enzyme Ply900

**Figure 6:**
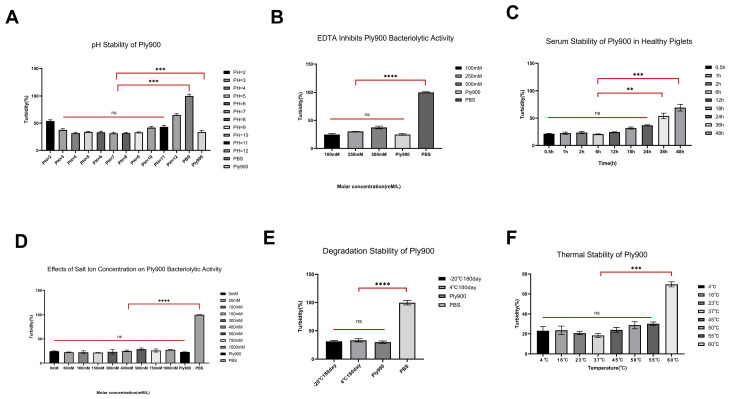
The biochemical properties of bacteriophage endolysin Ply900 were evaluated using *S. suis* SC19. Ply900 group: Untreated lysin Ply900; PBS group: No lysin Ply900. (**A**) Stability of Ply900 at varied pH. (**B**) Stability of Ply900 in the presence of EDTA. (**C**) Stability of Ply900 in serum from healthy piglets. (**D**) Salt-ion stability of Ply900. (**E**) Degradation stability of Ply900; differences between groups were assessed using Student’s *t*-test. (**F**) Thermal stability of Ply900. All experiments were replicated three times; error bars denote SD. (ns *p* > 0.05, ** *p* < 0.01, *** *p* < 0.001, **** *p* < 0.0001).

The bacteriophage lytic enzyme Ply900 exhibited good lytic activity within the temperature range of 4–55 °C, with peak activity occurring at 37 °C. Above 37 °C, its activity gradually decreased with increasing temperature and declined significantly at 60 °C (Figure 6F). This enzyme exhibited high cleavage activity within the pH range of 3.0–11.0, peaking at pH 7.0, but showing reduced activity at pH 2.0 and 12.0 (Figure 6A). Control reactions in the absence of enzyme demonstrated variations of ≤0.12 pH units throughout the incubation period. Furthermore, the cleavage activity of Ply900 remained largely unaffected at the salt concentrations ranging from 0.1 to 1.0 M (Figure 6D), and Ply900 demonstrated tolerance to 0.5 M EDTA (Figure 6B). The cleavage efficiency remained favorable during incubation in piglet serum at 37 °C for 0 to 24 h but declined significantly after 36 h (Figure 6C). After storage at 4 °C for 180 days, no reduction in Ply900 cleavage activity was observed (Figure 6E).

### 3.4. Effects of the Bacteriophage Lytic Enzyme Ply900 on Biofilms

**Figure 7:**
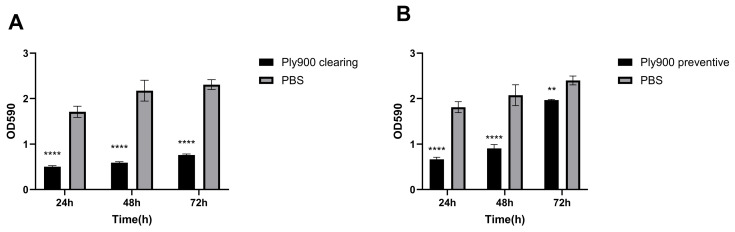
(**A**) Ply900 removes biofilms formed by *S. suis* SS9 at 24 h, 48 h, and 72 h. (**B**) Ply900 inhibits biofilm formation by *S. suis* SS9 at 24 h, 48 h, and 72 h. Crystal-violet staining quantified biofilm biomass (OD590) at 24 h, 48 h, and 72 h. Significant differences were observed between the Ply900 group and the PBS group (** *p* < 0.01, **** *p* < 0.0001). All experiments were replicated three times; error bars denote SD.

A semiquantitative assessment of biofilm formation, based on crystal violet staining, indicated that 20 μg/mL Ply900 effectively disrupted mature biofilms and significantly inhibited biofilm formation at both 24 and 48 h. Still, some inhibitory effects were noted on the 72 h biofilms, albeit with diminished efficacy (Figure 7A,B).

### 3.5. Ply900 Protects Mice Against S. suis Serotype SC19 Infection

**Figure 8:**
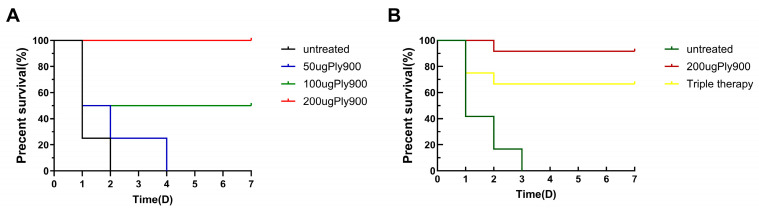
(**A**) In vitro minimum protein protective dose assay. Four groups of 4 animals each, Log-rank test (overall): χ^2^ = 10.46, df = 3, *p* = 0.015; dose-dependent trend: χ^2^ = 10.32, df = 1, *p* = 0.0013. (**B**) Survival rates of mice protected by Ply900 against lethal *S. suis* SC19 infection under different treatment regimens. Four groups of 24 animals each, Kaplan–Meier curves show significantly improved survival with treatments vs. untreated control (log-rank test: χ^2^(2) = 24.12, *p* = 0.0011). • Untreated: median 1 day (95% CI: 1–2) • 200ugPly900: NR at endpoint (>50% survival) • Triple therapy: NR at endpoint (>50% survival).

Mice infected with SC19 at a lethal dose (8 × 10^8^ CFU) developed systemic bacteremia within 1 h post-infection, exhibiting bloodstream bacterial loads of 1 × 10^5^ CFU/mL. Mice subjected to treatment with Ply900 at doses of 50 μg, 100 μg, and 200 μg per mouse exhibited survival rates of 20%, 80%, and 100%, respectively. Ultimately, 200 μg of Ply900 was identified as the most effective therapeutic dose tested under the experimental conditions employed (Figure 8A).

**Figure 9:**
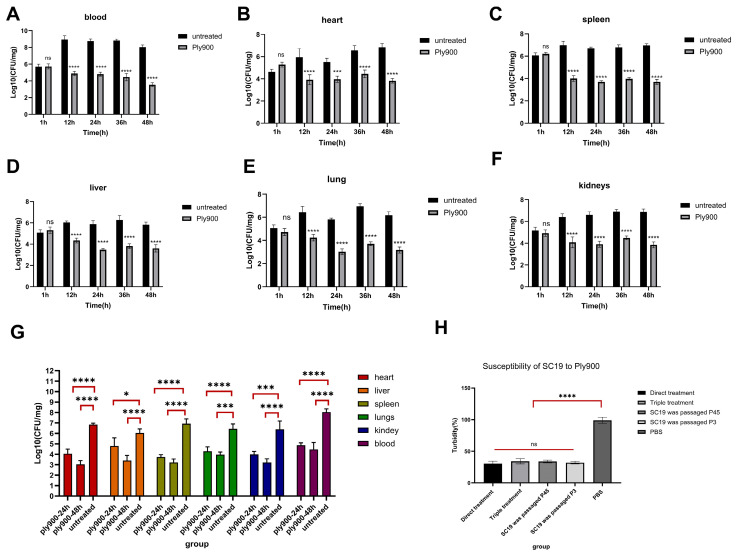
Protection by direct treatment with Ply900 against lethal porcine *S. suis* SC19 infection in various organs. (**A**) Blood bacterial load. (**B**) Cardiac bacterial load. (**C**) Splenic bacterial load. (**D**) Liver bacterial load. (**E**) Lung bacterial load. (**F**) Kidney bacterial load. (**G**) Ply900 triple therapy for treating haemorrhagic septicaemia caused by *S. suis* SC19. (**H**) Susceptibility of SC19 to Ply900. Significant differences in organ and blood bacterial loads were assessed using two-way analysis of variance (ANOVA) and implemented Holm–Šidák multiple-testing correction. (ns *p* > 0.05, * *p* < 0.05, *** *p* < 0.001, **** *p* < 0.0001). All experiments were replicated three times; Data are expressed as mean ± SD.

The survival rate in the direct treatment group was 91.7%, whereas that in the control group was 0%. The survival rate in the triple therapy group was 66.7%, whereas that in the control group was 0% (Figure 8B). In vitro assays confirmed that *S. suis* strains surviving in mice—whether from direct Ply900 treatment or triple therapy groups—retained full susceptibility to the bacteriophage lytic enzyme Ply900. Critically, even after 45 successive passages, SC19 remained susceptible to Ply900 (Figure 9H). Furthermore, the Minimal Effective Concentration of the enzyme in vitro remained essentially consistent with that of the original *S. suis* strains. Mice surviving 48 h after treatment in both direct treatment and triple therapy groups demonstrated favorable prognoses.

The therapeutic potential of Ply900 in a mouse model was evaluated by comparing bacterial loads in the blood and organs (heart, lungs, liver, spleen, and kidneys), inflammatory cytokine changes, and histopathological assessments among the direct treatment group, triple therapy group, and control group following infection with lethal doses of *S. suis* SC19. One hour after challenge with the lethal dose, mice in all groups developed systemic infection, with blood bacterial loads exceeding 10^6^ CFU/mL (Figure 9A). In the control group, the blood bacterial load peaked at 10^9^ CFU/mL within 12 h (Figure 9A). The cardiac and hepatic bacterial loads reached about 10^6^ CFU/mg at 12 h, declined slightly by 24 h, and then rebounded (Figure 9B,D), whereas the spleen, kidney, and lung loads reached maximum levels of about 10^7^ CFU/mg at 12 and 36 h, respectively (Figure 9C,E,F). All the PBS-treated control mice died within 48 h.

After systemic infection with *S. suis* serotype 2, mice in the direct treatment group that were administered 200 μg/mouse (Ply900) through direct injection presented significantly reduced bacterial loads in the blood and organs after 12 h. Compared to the control group mice without Ply900 treatment, the bacterial loads decreased by 1.16, 1.36, 1.13, 2.2, 0.52, and 0.9 log10 values, respectively. Compared to those in the control group at the same time point, the reductions were 4.04, 2.05, 2.2, 2.15, 2.9, and 2.3 log10 values, respectively (Figure 9). In the direct treatment group, the blood and organ bacterial loads progressively decreased from 12 h to 48 h. The organ bacterial loads rose slightly at 36 h before declining again, reaching the lowest values at 48 h. Compared to the control group, the triple therapy group presented significantly lower bacterial loads in the blood and organs, reaching the lowest levels at 48 h post-treatment. The bacterial loads in the blood, heart, liver, spleen, lungs, and kidneys decreased by 4.4, 3.84, 2.7, 3.7, 2.5, 3.1, and 4.4 log10 values, respectively, relative to those in the control group (Figure 9).

**Figure 10:**
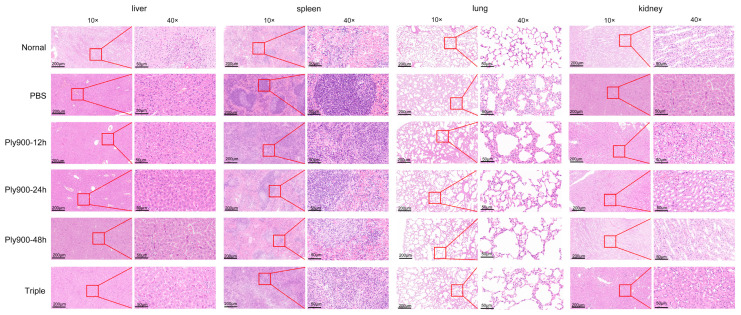
Histopathological analysis.

Histological assessment revealed varying degrees of damage to different organs, including the heart, liver, spleen, lungs, and kidneys, in the control mice not treated with the bacteriophage lytic enzyme Ply900. In untreated mice, progressive pulmonary lesions exhibited marked congestion and hemorrhage throughout the disease course. Alveolar capillary dilation and congestion were observed along with thickened alveolar walls and inflammatory cell infiltration within the alveolar spaces. Congestion and hemorrhage were observed in the venous sinuses of the red pulp of the spleen. Lymphocytic necrosis, accompanied by a small number of neutrophilic infiltrates, was also observed. Congestion of hepatic vessels was noted, with minor red blood cell stasis visible between the hepatic cords. Minimal neutrophilic infiltration was present in the lower regions of the renal lesions. Compared to those in the triple therapy group, the histopathological lesions in the mice in the direct treatment group treated with the bacteriophage lytic enzyme Ply900 were markedly reduced. No significant histopathological lesions were observed in the blank control group (Figure 10).

**Figure 11:**
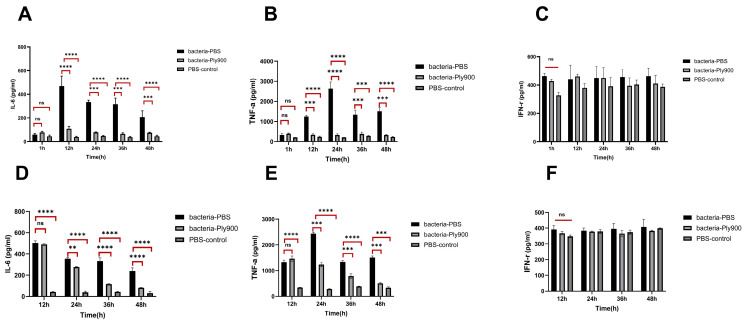
Protection of inflammatory cytokine levels in mice infected with lethal-dose *S. suis* SC19 by different Ply900 treatment regimens. (**A**) IL-6 levels at different time points following direct Ply900 treatment. (**B**) TNF-α levels at different time points following direct Ply900 treatment. (**C**) IFN-γ levels at different time points following direct Ply900 treatment. (**D**) IL-6 levels at different time points following triple therapy with Ply900. (**E**) TNF-α levels at different time points following triple therapy with Ply900. (**F**) IFN-γ levels at different time points following triple therapy with Ply900. Differences between the direct Ply900 treatment group, triple treatment group, and PBS control group were assessed using two-way analysis of variance (ANOVA)and implemented Holm–Šidák multiple-testing correction. (ns *p* > 0.05, ** *p* < 0.01, *** *p* < 0.001, **** *p* < 0.0001). All experiments were replicated three times; error bars denote SD.

Compared to the blank group, the control group showed a significant upregulation of the pro-inflammatory cytokines IL-6 and TNF-α at 12 h post-infection. The concentrations of TNF-α and IL-6 reached peak levels of 470.0 pg/mL and 475.8 pg/mL, respectively. In the period between 12 and 48 h post-infection, the concentrations of the pro-inflammatory cytokines IL-6 and TNF-α slightly decreased over time. The direct treatment group and triple therapy group presented significantly lower IL-6 and TNF-α levels than did the control group between 12 h and 48 h (Figure 11A–E). Furthermore, the levels of the pro-inflammatory cytokines IL-6 and TNF-α in the triple therapy group were marginally higher than those in the direct treatment group. Within 48 h post-infection, the IFN-γ levels in the control group remained low, with no significant differences observed between the direct treatment group, triple therapy group, control group, and blank group (Figure 11C,F).

## 4. Discussion

*S. suis* infection is a critical health threat to the global swine industry. Notably, the inappropriate use and overreliance on antibiotics have led to a year-over-year surge in the prevalence of MDR strains, with serotype 2 being the most prominent and concerning among all of them [16,17]. Antibiotic therapy remains the primary treatment option for *S. suis* infections at present [18]. Bacteriophage lytic enzymes, due to their precise targeting of bacterial peptidoglycan and their significant reduction in the risk of developing resistance, represent one of the key alternative approaches for overcoming the challenge of antibacterial resistance [19,20].

In this study, the phage lytic enzyme Ply900 isolated from the pre-phage of *Streptococcus parasuis* was evaluated and found to possess a broader lytic spectrum and higher lytic efficiency. The enzyme was capable of specifically lysing *S. suis*, *S. agalactiae*, and *S. aureus*. The active substance effectively inhibited and cleared the biofilms formed by the target bacteria after 24 h and 48 h, significantly reducing the number of fixed bacteria within the biofilm. The minimal effective concentration of the enzyme in vitro was determined to be 1 µg/mL. The enzyme activity of Ply900 remained stable within a pH range of 3.0–11.0 and at temperatures ≤ 55 °C. This property significantly outperforms that of the several other reported streptococcal lytases, such as LytA, which exhibits > 60% loss of activity above 40 °C and inactivation at pH < 5.0, demonstrating the potential of the enzyme in this study to withstand the complex microenvironments within the host organism [21].

The bacteriophage lytic enzyme Ply900 comprised a CD (CHAP) cleavage domain and a CBD (SH3B) cell wall-binding domain linked by a flexible spacer (G-G-S)3, which enables this enzyme to slide along the peptidoglycan surface while scanning its substrate [22,23]. The CHAP domain of the *S. suis* bacteriophage specifically recognized the D-glutamine (D-Glu)-L-lysine (L-Lys) cross-link in the peptidoglycan of *S. suis*. In contrast, the SH3B domain could selectively bind the pentaglycine bridge within the peptidoglycan. The SH3B domain anchored the CHAP domain to the surface of the peptidoglycan layer, thereby significantly increasing the local enzyme concentration and enhancing substrate contact efficiency [24,25]. Truncation experiments demonstrated that the CHAP domain alone possessed cleavage activity, albeit slightly lower than that of the full enzyme. The full-length Ply900 and SH3B domains could bind to *S. suis* cell walls but not to those of *E. coli*. The CHAP domain had an α/β-folded structure with a core double β-barrel conformation, containing the conserved Cys-His-Glu/Asn catalytic triad [26,27]. Point mutations introduced at GLU-59, HIS-28, and CYS-34 within the CHAP domain of Ply900d resulted in a reduction of over 90% in the antibacterial activity of Ply900, confirming that the Cys-His-Glu catalytic triad constitutes the critical amino acid site for the effect of the Ply900 CHAP lytic domain. After the high-exposure Asp86 mutation, the lysis efficiency decreased by about 40%. Owing to its position at the N-terminal substrate channel entrance of the CHAP domain, the mutation may have altered the channel’s electrostatic barrier, thereby affecting the substrate entry kinetics [27].

A *S. suis* type 2 infection model was established in this study to evaluate the in vivo therapeutic efficacy of Ply900 as an antibacterial agent. After systemic infection, intraperitoneal injection of 200 μg/mouse Ply900 yielded a survival rate of 91.7% (11/12). The protease Ply30 at 2 mg/mouse protected 80% of the mice from death after systemic infection with type 2 HA9801 *S. suis* [28]. PLY5218 at 200 μg/mouse protected 90% of mice from death following systemic infection with type 2 HA9801 *S. suis* [29], these data demonstrate that Ply900 exhibits superior efficacy and therapeutic effects, achieving a higher survival rate at the same dose compared with PLY5218, and demonstrating a far better dose-efficacy ratio than Ply30. The triple therapy regimen yielded a mouse survival rate of 66.7% (8/12), with 75% of the deceased mice dying prior to administration. Direct therapy is reported to be markedly superior to triple therapy, primarily owing to its administration shortly after bacterial invasion, thereby achieving effective suppression of local infection and bacterial dissemination [30]. In the control group, the levels of the inflammatory cytokines IL-6 and TNF-α were significantly increased, whereas those in the direct treatment group and triple therapy group remained relatively stable. Furthermore, the residual *S. suis* bacteria that were not eliminated and remained within the mice were sensitive to the lytic enzyme Ply900, effectively preventing the induction of bacterial resistance gene expression.

Compared to those in the control group, the bacterial loads in the blood and organs (heart, liver, spleen, lungs, kidneys) were significantly lower 48 h after direct therapy and triple therapy. Notably, despite the discussion of organ-related clearance processes, pharmacokinetic data were not assessed in this study. Notably, marked pathological damage was observed in the lungs and spleen of the mice: in the lungs, alveolar capillary dilation and congestion, thickened alveolar walls, and inflammatory cell infiltration in the alveolar spaces were evident; in the spleen, congestion and hemorrhage occurred in the venous sinuses of the red pulp, accompanied by lymphocytic necrosis and a small number of neutrophilic infiltrates. This reflects a key manifestation of the organ-specific tissue adaptation strategy evolved by *S. suis* serotype 2 [30,31,32]. Within the lungs, SS2 surface adhesins directly undermined the tight junctions in the alveolar epithelium by binding with high affinity to the basement membrane-associated mucin, leading to a marked increase in vascular permeability. Bacteria-secreted lysozyme forms β-barrel pores in alveolar cell membranes, inducing cell lysis and diffuse hemorrhage [31]. Within the spleen, the capsular polysaccharide CPS2 effectively inhibits complement C3b deposition, reducing the phagocytosis rates by 90% [33], thereby enabling bacterial escape from immune clearance. Meanwhile, SS2 invading the splenic macrophages activates the NLRP3 inflammasome pathway through Sly, triggering gasdermin D pore formation and achieving a pyroptosis rate of 68.3% [34,35], leading to the disintegration of the splenic tissue architecture. *S. suis* serotype 2 achieves this goal by precisely targeting the pulmonary tissue for adhesion and colonization while simultaneously undermining the core immune defenses of the spleen, thereby causing severe pathological damage throughout the organism [32,35].

Theoretically, pre–injection of lysozyme can effectively prevent bacterial infection, provided that the lysozyme maintains its basic lytic activity within the target animal for a certain period. However, proteins smaller than 60 kDa are often rapidly eliminated by the renal system, and this study further lacks data on Ply900’s toxicity, immunogenicity, PK/PD, and residue depletion—gaps to be addressed in future research for full translational validation. The in vitro experiments conducted with Ply900 in this study demonstrated a significant reduction in its lytic activity following 36 h of incubation in serum. Consequently, it was inferred that Ply900 may prove incapable of providing sustained efficacy against *S. suis* infections. Notably, it was tolerant to 1 M NaCl and 0.5 M EDTA (with >90% activity retention), indicating that the enzyme can retain its antibacterial function even at the infection sites containing metal ions or high osmotic pressure. Furthermore, residual *S. suis*, which was not eliminated and remained in mice, was susceptible to the lytic enzyme Ply900, effectively preventing the induction of bacterial resistance gene expression. These findings collectively establish crucial theoretical foundations for broadening the in vivo applications of Ply900. Collectively, these findings establish a preliminary foundation for the potential application of the protease Ply900 in addressing *S. suis* infections.

In summary, this study identified a novel *S. suis* bacteriophage lytic enzyme, Ply900, which exhibits lytic activity against various serotypes of Streptococcus in vitro. In the in vivo experiments, this enzyme exhibits protective effects in the mice challenged with lethal doses of *S. suis* serotype 2. Regarding therapeutic efficacy, Ply900 should be administered shortly after infection or at increased doses to provide stronger protection. The experimental results of this study suggest that lysozyme Ply900 can be considered a potential antimicrobial agent capable of effectively treating *S. suis* type 2 infections, demonstrating significant potential as an alternative to antibiotics.

## Figures and Tables

**Table 1:** Minimal Effective Concentration (MEC) of Ply900.

Strain	Species of Strain	MEC (µg/mL)
SS-XS-0507	*S. suis* (serotype 1)	5
SS-XA-0507	*S. suis* (serotype 1)	10
SS-SC-1024	*S. suis* (serotype 2)	5
SS-MF-0001	*S. suis* (serotype 2)	5
SS-SC-0921	*S. suis* (serotype 2)	5
SS-SC19	*S. suis* (serotype 2)	10
SS-BX-1012	*S. suis* (serotype 3)	1
SS-BX-1029	*S. suis* (serotype 3)	5
SS-ZZ-0001	*S. suis* (serotype 4)	2.5
SS-HG-0001	*S. suis* (serotype 4)	5
SS-ZZ-0209	*S. suis* (serotype 4)	5
SS-FY-0723	*S. suis* (serotype 7)	2.5
SS-FY-L	*S. suis* (serotype 7)	5
SS-LJ-0026	*S. suis* (serotype 7)	5
SS-SY-0609	*S. suis* (serotype 7)	10
SS-BX-0717	*S. suis* (serotype 8)	10
SS-NA-1006	*S. suis* (serotype 9)	1
SS-BX-1220	*S. suis* (serotype 9)	1
SS-BX-P	*S. suis* (serotype 9)	5
SS-XL-0001	*S. suis* (serotype 12)	5
SS-BX-L	*S. suis* (serotype 18)	2.5
SS-BZ-0001	*S. suis* (serotype NT)	1
SS-YL-0001	*S. suis* (serotype NT)	1
SS-LZ-0001	*S. suis* (serotype NT)	2.5
SS-FY-0925	*S. suis* (serotype NT)	2.5
SS-SC-0923	*S. suis* (serotype NT)	5
SS-YL-1101	*S. suis* (serotype NT)	5
SS-LJ-1007	*S. suis* (serotype NT)	5
SS-BZ-0427	*S. suis* (serotype NT)	5
SS-JJ-1024	*S. suis* (serotype NT)	5
SS-HG-1010	*Streptococcus parasuis*	1
SS-XL-0917	*S. suis* (serotype NT)	10
SA-WW-1	*S. agalactiae* (serotype III)	10
SA-WW-2	*S. agalactiae* (serotype III)	10
SA-WW-3	*S. agalactiae* (serotype III)	15
S. aureus-WW-1	*S. aureus* (capsular serotype 5)	15

## Data Availability

The original contributions presented in this study are included in the article. Further inquiries can be directed to the corresponding authors.

## Supplementary Materials

The following supporting information can be downloaded at: https://www.mdpi.com/article/10.3390/vetsci13010065/s1, Figure S1: The complete blot image.

## Abbreviations

The following abbreviations are used in this manuscript:

MDR	Multi-drug Resistance
MEC	Minimal Effective Concentration
*S. suis*	*Streptococcus suis*
OD600	optical density at 600 nm
PBS	Phosphate-Buffered Saline
