# Supplementary material for: Antibacterial Activity of Bacteriophage Lytic Enzyme Ply900
Source: Vet Sci. 2026 Jan 9;13(1):65. doi: 10.3390/vetsci13010065 (PMC12846548; doi:10.3390/vetsci13010065)
Supplement: Supplementary file 1 [file vetsci-13-00065-s001.zip › vetsci-4081716-supplementary.pdf]

1. Fundamental Characteristics of Phage Lysin Ply900

Gene ID :GE000900
Start :982,186
End :982,905
Length :720 bp
Nr annotation :CHAP domain-containing protein [Streptococcus suis]
GO annotation :Molecular Function: hydrolase activity (GO:0016787)
TrEMBL annotation :Cell wall hydrolase
eggNOG annotation :NOG111849 domain protein [S]: Function unknown [S]
Pfam annotation :PF08460;PF05257 SH3_5;CHAP SH3 domain;domain

CDS sequences：
ATGACAACAGTAAATGAAGTAGTTAATTTTGCCAAAGACCTTGCCAATCGT
GGTCAAGGTGTAGACTATGATGGTTGGTACGGTAATCAATGTGTAGACCTAC
CTAACTGGATTTGTGGCAAGTTTTTCGGCAAAGCTCTTTGGGGCAACGCCA
TTGATTTGATTAAATCAGCAAAGCGACACGGATTCGAGGTGCATTATATGCC
TACCTCAGAACGTCCACGTCCAGGGGCTATCTTTGTCAAGAATTACTGGGC
AGGTGACGGTATCAACTATGGGCATACTGGTCTGATTATCGGAGTCAGTGG
CAATACTGTCCAAACTATTGAGCAAAATCTTGTTGGTAATTTGTCGGTCGGT
GGACCTGCTCAATATGCTAGCCAGCAAATCAGCAATCTTGTTGGCTGGTTTT
ATCCACCTTACAGCGACTCTACTGCAGTGGCAACACAGGCAAGCAGTGGC
AATCTCGGTAAGGTCAAAGACGAGAAGGGGACAATGACCGTTAAAGTATC
TTTGCTCAATGTCCGAGACAAGCCTGGTCTAGACGGTAAAGTTGTGGCAAC
GTACACGAATGGCGAGCAGTTTAATTATGATTCGGTCTATATTGCCGATGGAT
ACATTTGGGTATCGTATGTTAGTCGTAGCGGTGTACGTCGCTATGTAGCAGC
AGGCGAGGAGTCAAATCGACGCAATGTCGTGCCTTACGGTACGTTTAAATA
G

Protein sequences：
MTTVNEVVNFAKDLANRGQGVDYDGWYGNQCVDLPNWICGKFFGKALWG
NAIDLIKSAKRHGFEVHYMPTSERPRPGAIFVKNYWAGDGINYGHTGLIIGVS
GNTVQTIEQNLVGNLSVGGPAQYASQQISNLVGWFYPPYSDSTAVATQASSGN
LGKVKDEKGTMTVKVSLLNVRDKPGLDGKVVATYTNGEQFNYDSVYIADGY
IWVSYVSRSGVRRYVAAGEESNRRNVVPYGTFK*

2. Homology Model Template for Modeling Phage Lysin Ply900

The three-dimensional (3D) structure of the phage lysin Ply900 was predicted using the homology modeling approach. The template structure selected for this modeling was the N-acetylmuramoyl-L-alanine amidase from *Streptococcus suis* (Gene: *lytA*), retrieved from the AlphaFold Database (Entry:A0A075C014.1.A).

N-acetylmuramoyl-L-alanine amidase, Sequence length 239

MTTVNEVVNFAKDLANRGQGVDYDGWYGNQCVDLPNWICGKFFGKPLWG
NAIDLIKSAKQHGFEVHYMPTSERPRPGAIFVKNYWASDGVNYGHTGLIIGVS
GNAVQTIEQNLVGNLSVGGPAQYASQQISNLVGWFYPPYSDSTAVATQASSGN
LGKYKDEQGTMTVKVSLLNVRDKPSLDGKIVATYTYSEQFNYDSIYIADGYF
WVSYVSRSGVRRYVAAGEESNRRNVVPYGTFK

In summary, the Ply900 homology model was built using a template with exceptionally high sequence identity (94.56%), a high intrinsic quality score (GMQE=0.85), and direct functional relevance. Subsequent rigorous model quality assessments confirm that the final model possesses excellent stereochemical properties. Therefore, we are confident that this model is highly reliable for subsequent analyses, including catalytic site characterization, mechanistic studies, and molecular docking simulations.

Exceptional Sequence Identity: A pairwise sequence alignment between Ply900 and the template revealed a sequence identity of 94.56%. A sequence identity above 30% is generally considered sufficient for reliable homology modeling. A value exceeding 94% indicates that the two proteins are highly likely to share an identical fold, making the template exceptionally suitable for generating a high-accuracy model.

High Predicted Model Quality: The Global Model Quality Estimation (GMQE) score provided by the SWISS-MODEL server was 0.85. The GMQE score is a composite reliability score that combines properties from the target-template alignment and the template structure. It ranges from 0 to 1, with higher scores indicating higher expected accuracy. A score of 0.85 signifies a high-quality and reliable model is anticipated.

Functional Relevance: The template protein is a well-characterized bacterial autolysin. Its catalytic activity, the hydrolysis of the amide bond between N-acetylmuramoyl and L-alanine residues in bacterial cell wall peptidoglycan, is identical to the expected function of the phage lysin Ply900. This functional congruence strongly supports the biological relevance of the chosen template.

3. Supplementary Figure S1

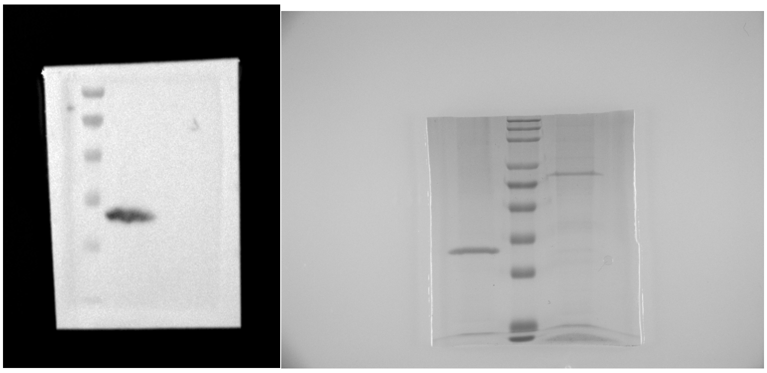

Figure S1: The complete blot image.
